# Identification of lateral pelvic nodes without metastasis in patients with rectal cancer treated with preoperative chemoradiotherapy or chemotherapy based on magnetic resonance imaging

**DOI:** 10.1002/ags3.12832

**Published:** 2024-06-01

**Authors:** Nobuaki Hoshino, Yudai Fukui, Kohei Ueno, Koya Hida, Kazutaka Obama, Kazuhiro Sakamoto, Hirotoshi Kobayashi, Michio Itabashi, Soichiro Ishihara, Kazushige Kawai, Yoichi Ajioka

**Affiliations:** ^1^ Department of Surgery Kyoto University Graduate School of Medicine Kyoto Japan; ^2^ Department of Gastroenterological Surgery Toranomon Hospital Tokyo Japan; ^3^ Department of Coloproctological Surgery, Faculty of Medicine Juntendo University Tokyo Japan; ^4^ Department of Surgery Teikyo University Hospital Mizonokuchi Kanagawa Japan; ^5^ Department of Surgery Institute of Gastroenterology Tokyo Women's Medical University Tokyo Japan; ^6^ Department of Surgical Oncology The University of Tokyo Tokyo Japan; ^7^ Department of Colorectal Surgery Tokyo Metropolitan Cancer and Infectious Diseases Center Komagome Hospital Tokyo Japan; ^8^ Division of Molecular and Diagnostic Pathology, Graduate School of Medical and Dental Sciences Niigata University Niigata Japan

**Keywords:** chemoradiotherapy, lateral pelvic node, magnetic resonance imaging, neoadjuvant chemotherapy, rectal neoplasms

## Abstract

**Background:**

Intensive localized therapy is promising for the treatment of rectal cancer. In Japan, chemoradiotherapy (CRT) and neoadjuvant chemotherapy (NAC) are used as preoperative treatments for this disease. Magnetic resonance imaging (MRI) is used to diagnose lateral pelvic node (LPN) metastases, but the changes in LPN findings on MRI following preoperative treatment are unclear. Furthermore, there may be patients in whom LPN dissection can be omitted after CRT/NAC.

**Methods:**

Patients who underwent total mesorectal excision with LPN dissection after CRT/NAC at 13 Japanese Society for Cancer of the Colon and Rectum member institutions between 2017 and 2019 were included. Changes in the short diameter of the LPNs after CRT/NAC and the reduction rate were examined.

**Results:**

A total of 101 LPNs were examined in 28 patients who received CRT and 228 in 47 patients who received NAC. Comparison of LPNs before and after CRT/NAC showed that most LPNs shrank after CRT but that the size reduction was variable after NAC. Although some LPNs with a short diameter of <5 mm showed residual metastasis, no metastases were observed in LPNs that were <5 mm in short diameter before and after CRT/NAC and did not shrink after treatment.

**Conclusion:**

Although the short diameter of LPNs was significantly reduced by both CRT and NAC, even LPNs with a short diameter of <5 mm could have residual metastases. However, dissection may be omitted for LPNs <5 mm in short diameter that do not shrink after preoperative CRT or NAC.

## Graphical abstract

Both chemoradiotherapy (CRT) and neoadjuvant chemotherapy (NAC) significantly reduced the short diameter of lateral pelvic nodes (LPNs), but even LPNs with a short diameter of <5 mm could contain residual metastases in patients with rectal cancer. LPNs with a short diameter of <5 mm both before and after CRT or NAC that did not shrink after treatment showed no evidence of metastasis, suggesting that LPN dissection could be omitted in these cases.

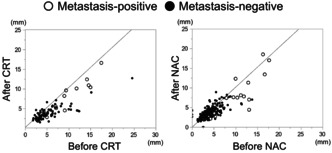

## 1 INTRODUCTION

Prevention of local recurrence is important in the treatment of advanced rectal cancer.^1^, ^2^, ^3^, ^4^ Local recurrence in patients with advanced rectal cancer is estimated to occur at a rate of 7%–18% and is extremely difficult to cure.^5^, ^6^ Lateral pelvic node (LPN) dissection is often performed for advanced rectal cancer in Japan and other Asian countries, while preoperative chemoradiotherapy (CRT) is used in Western countries. Neither treatment alone is considered effective enough to prevent local recurrence.^7^, ^8^, ^9^ In recent years, intensive local treatment that combines both of those treatments has been attracting interest.^10^, ^11^, ^12^ Although such intensive treatment appears to be promising, there is no consensus on the indications for LPN dissection in patients who are treated preoperatively.

Magnetic resonance imaging (MRI) is the modality most frequently used for diagnosis of LPN metastases. In terms of diagnostic accuracy, MRI has been reported to have a sensitivity of 90%–100% and a specificity of 14%–45%.^13^ Several risk factors for LPN metastasis have been identified, including the LPN short diameter and extramural vascular invasion, and prediction models for diagnosis of LPN metastasis that include these factors have been reported.^14^, ^15^, ^16^, ^17^ The short diameter, long diameter, internal structure, and margin status of LPNs, determined using the shape of LPNs as seen on MRI, are considered useful for diagnosing metastases, with the short diameter of the LPNs being the most widely used.^18^

In this study, we focused on the short diameter of LPNs as seen on MRI both before and after preoperative treatment with the aim of identifying patients with rectal cancer in whom LPN dissection could be omitted by detecting the features of LPN without residual metastasis after preoperative treatment.

## 2 PATIENTS AND METHODS

### 2.1 Study design and setting

Data on patients who underwent total mesorectal excision and LPN dissection for rectal cancer between January 2017 and December 2019 were collected prospectively by the Japanese Society for Cancer of the Colon and Rectum MRI Study Group, which includes 13 affiliated referral hospitals. Details of this study are described in an earlier report by the MRI Study Group.^13^ Patients who received CRT or neoadjuvant chemotherapy (NAC) were extracted and LPNs were identified on MRI scans obtained before or after CRT/NAC. The presence or absence of residual metastasis in LPNs was determined by pathological diagnosis after LPN dissection.

MRI was performed under the following conditions: (1) an MRI scanner of 1.5 T or higher was used; (2) images in two or more directions were taken in 3 mm slices in the primary lesion and lateral regions; (3) contrast‐enhanced MRI was performed with T2 enhancement without fat suppression; 4) MRI imaging after preoperative treatment was performed within 1 month before surgical treatment.

The diagnostic performance for LPN metastasis based on the short diameter of LPNs on MRI before and after CRT or NAC was investigated. We also examined changes in the short diameter of LPNs after preoperative treatment and compared them between the CRT group and the NAC group. Furthermore, we attempted to develop diagnostic criteria for LPNs without residual metastases to identify patients in whom LPN dissection could be omitted after CRT or NAC.

### 2.2 Statistical analysis

The diagnostic performance of MRI was investigated by analysis of the area under the curve (AUC). Changes in the short diameter of the LPNs after CRT or NAC were plotted and assessed visually according to the presence or absence of residual LPN metastasis. Changes in the short diameter of LPNs after CRT or NAC were examined separately for residual LPN metastasis using the paired *t*‐test.

LPNs were divided into groups based on the short diameter seen before and after CRT or NAC (0–4.9 mm, 5–9.9 mm, ≥10 mm). The LPNs were also divided into groups based on the reduction rate of the short diameter after CRT or NAC (no reduction, 0.01–0.49, ≥0.50). The features of LPNs without residual metastasis were then investigated based on the short diameter and reduction rate. The relation between the short diameter of LPN and the reduction rate was examined by using the Fisher's exact test. LPNs that were no longer visible on MRI after preoperative treatment were considered to have shrunk due to treatment and their short diameter was treated as 0 mm.

## 3 RESULTS

### 3.1 Patient characteristics

**FIGURE 1:**
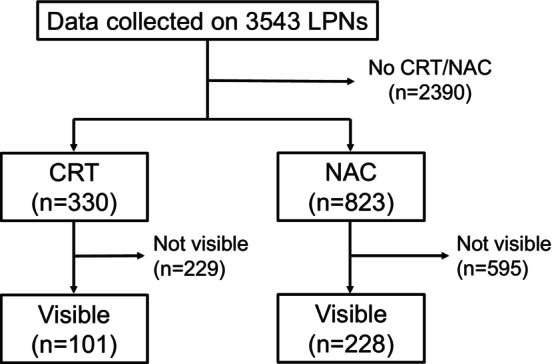
Flow diagram of lateral lymph node selection. CRT, chemoradiotherapy; LPNs, lateral pelvic nodes; NAC, neoadjuvant chemotherapy.

Data were collected by the MRI Study Group for 3543 LPNs (212 patients) identified by pathological examination. From these data, we extracted 330 LPNs from 28 patients who had received preoperative CRT and 823 LPNs from 47 patients who had received preoperative NAC. LPNs that could not be identified on MRI both before and after preoperative treatment were excluded. Finally, 101 LPNs from 28 patients in the CRT group and 228 LPNs from 47 patients in the NAC group were included in the study (Figure 1). The patient characteristics are shown in Table 1. The details of regimens about CRT and NAC were shown in Table S1.

**TABLE 1:** Patient characteristics.

Variable	CRT	NAC
	(*n* = 28)	(*n* = 47)
Age (years)^a^	58.5 (35–78)	58 (29–75)
Sex (male/female)	19/9	34/13
Comorbidity (no/yes)	9/19	34/13
Tumor location (upper rectum/lower rectum/anal canal)	0/23/5	4/35/8
Distance from anal verge (0/>0, ≤5/<5)	5/11/12	1/24/22
Pathological diagnosis (differentiated/undifferentiated)	25/3	47/0
CEA (<5/≥5)	12/16	24/23
cT (1/2/3/4a/4b)	0/0/21/2/5	1/1/30/3/12
cN (0/1/2/3)	2/1/1/24	13/11/7/16
cM (0/1)	26/2	43/4
Approach (open/laparoscopic/robotic‐assisted)	5/18/5	2/37/8
Surgical procedure (LAR/ISR/APR/other)	13/4/10/1	20/14/11/2
LPN dissection (unilateral/bilateral)	22/6	5/42
Combined resection (no/yes)	22/6	39/8
Operation time (min)^a^	455 (274–808)	522 (297–1185)
Estimated blood loss (mL)^a^	180 (30–1540)	130 (0–1260)

Abbreviations: APR, abdominoperineal resection; CEA, carcinoembryonic antigen; CRT, chemoradiotherapy; ISR, intersphincteric resection; LAR, low anterior resection; LPN, lateral pelvic node, NAC, neoadjuvant chemotherapy.

^a^ median, range.

### 3.2 Diagnostic performance of MRI

The diagnostic performance of MRI based on the short diameter of LPNs is shown in Table 2. The AUC for MRI was high at 0.86 both before and after treatment in the CRT group but was higher before treatment than after treatment in the NAC group (0.91 vs. 0.82).

**TABLE 2:** Diagnostic performance of magnetic resonance imaging determined by area under the curve according to type of preoperative treatment.

CRT		NAC	
Before CRT	After CRT	Before NAC	After NAC
0.86	0.86	0.91	0.82

Abbreviations: CRT, chemoradiotherapy; NAC, neoadjuvant chemotherapy.

### 3.3 Changes in short diameter of LPNs after preoperative treatment

**FIGURE 2:**
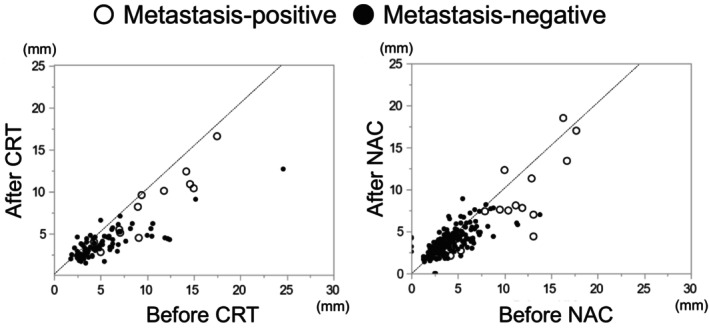
Changes in short diameter of lateral pelvic nodes before and after preoperative treatment. CRT, chemoradiotherapy; NAC, neoadjuvant chemotherapy.

The changes in the short diameter of LPNs after preoperative treatment are shown in Figure 2. Regardless of the presence or absence of residual metastases, the majority of LPNs shrank after CRT. After NAC, most LPNs with residual metastases shrank, while those without residual metastases varied in size from reduced, unchanged, to enlarged. Residual metastases were detected even in LPNs with a short diameter <5 mm. Furthermore, residual metastatic LPNs were observed in both groups even when the short diameter was reduced by preoperative treatment.

### 3.4 Reduction in short diameter of LPNs on MRI by preoperative treatment

Both CRT and NAC significantly reduced the short diameter of LPNs both with and without residual metastases (Table 3). However, NAC reduced the short diameter of LPNs without residual metastases very slightly (by 0.3 ± 1.4 mm).

**TABLE 3:** Reduction in short diameter of LPNs by preoperative treatment according to residual metastasis status.

Preoperative treatment	Length (mm)	LPNs without residual metastasis			LPNs with residual metastasis		
		Pre	Post	*p*‐value	Pre	Post	*p*‐value
CRT	Short diameter	5.2 (3.4)	3.8 (1.7)	<0.001	10.3 (4.3)	8.3 (4.1)	0.001
	Reduction	1.5 (2.2)			2.0 (1.6)		
NAC	Short diameter	4.0 (1.9)	3.7 (1.4)	0.003	10.5 (4.4)	8.3 (4.8)	0.004
	Reduction	0.3 (1.4)			2.2 (2.7)		

*Note*: Data are shown as the mean (standard deviation). Abbreviations: CRT, chemoradiotherapy; LPN, lateral pelvic node; NAC, neoadjuvant chemotherapy.

### 3.5 Identification of LPNs without residual metastasis after preoperative treatment

The distribution of LPNs according to residual metastasis status, short diameter of LPNs, and the reduction rate are shown for the CRT and NAC groups in Table 4. Even when the short diameter was <5 mm, residual metastasis was detected in 4.8% (3/62) of LPNs after CRT and 5.3% (5/95) after NAC. None of the LPNs with a short diameter of 0–4.9 mm that did not shrink after preoperative treatment with CRT or NAC showed residual metastasis. There was a significant association between the short diameter of LPN before preoperative treatment and the reduction rate in LPNs without residual metastases, but not in LPNs with residual metastases. The significant association between the short diameter of LPN and the reduction rate disappeared after preoperative treatment. There was a significant difference between LPNs with a short diameter <5 mm that had been shrunk by preoperative treatment and other LPNs in the NAC group (*p* < 0.001), but no significant difference in the CRT group (*p* = 0.205).

**TABLE 4:** Distribution of LPNs according to residual metastasis status, short diameter of LPNs, and reduction rate.

Short diameter of LPN (mm)	CRT						NAC					
	LPNs without residual metastasis			LPNs with residual metastasis			LPNs without residual metastasis			LPNs with residual metastasis		
	Reduction rate			Reduction rate			Reduction rate			Reduction rate		
	‐	0.01–0.49	0.5+	‐	0.01–0.49	0.5+	‐	0.01–0.49	0.5+	‐	0.01–0.49	0.5+
	Before CRT						Before NAC					
0–4.9	16	36	1	0	1	0	95	59	4	0	2	1
5–9.9	2	23	2	1	4	1	9	38	3	0	3	0
10+	0	4	5	0	5	0	0	3	0	2	8	1
	*p* < 0.001			*p* = 1.000			*p* < 0.001			*p* = 0.861		
	After CRT						After NAC					
0–4.9	16	51	8	0	2	1	88	83	7	0	3	2
5–9.9	2	11	0	1	3	0	16	17	0	0	7	0
10+	0	1	0	0	5	0	0	0	0	2	3	0
	*p* = 0.693			*p* = 0.318			*p* = 0.732			*p* = 0.018		

Abbreviations: CRT, chemoradiotherapy; LPN, lateral pelvic node; NAC, neoadjuvant chemotherapy.

## 4 DISCUSSION

In this study, the short diameter of LPNs was reduced in both the CRT and NAC groups. However, LPNs without metastasis after preoperative treatment shrank only slightly in the NAC group. Residual metastases in LPNs were found in both the CRT and NAC groups even when the short diameter was <5 mm but not in LPNs with a diameter <5 mm that had not been shrunk by CRT or NAC.

Intensive local treatment comprising total mesorectal excision plus LPN dissection followed by CRT or NAC is now becoming widely used at leading medical institutions in Japan and Korea.^19^, ^20^ It has been reported that local recurrence rates have been reduced to as low as 5% by intensive local treatment.^19^ However, the benefit of LPN dissection in terms of improving the long‐term prognosis has not been fully demonstrated, and an increase in postoperative complications such as urinary and sexual dysfunction has been reported.^21^, ^22^, ^23^, ^24^, ^25^, ^26^, ^27^, ^28^ In this study, we aimed to identify features of LPNs without residual metastasis in patients with rectal cancer who have received preoperative CRT or NAC in order to identify those in whom LPN dissection can be omitted. Although several features can be used to diagnose LPN metastasis on MRI, only the short diameter was used in this study for the sake of simplicity and ease of use.

Chemotherapy and chemoradiotherapy act more strongly on tumor cells than on normal cells and preoperative treatment is expected to shrink LPNs with metastases more than those without metastases.^29^ In the LPNs without residual metastases, there was a significant association between the short diameter of LPN and the reduction rate before preoperative treatment, but not after preoperative treatment. This was thought to be due to the shrinkage of large LPNs, which was caused by the disappearance of tumor cells after preoperative treatment. We focused on it and tried to establish criteria for LPNs free of metastases by using the short diameter of LPNs and their reduction rate. Because LPNs that had shrunk due to preoperative treatment were more likely to have tumor cells before preoperative treatment, we considered that LPNs that had not shrunk due to preoperative treatment were more likely to be free of metastasis. Also, we considered that larger LPNs might have residual tumor cells after preoperative treatment. Therefore, we thought that smaller LPNs that had not been shrunk by preoperative treatment would not show metastasis. In fact, among LPNs that had not shrunk due to preoperative treatment, no metastasis was found in LPNs with a short diameter of <5 mm.

MRI is the modality most frequently used for diagnosis of LPN metastasis in patients with rectal cancer. In a previous study, we sought to identify high‐performance diagnostic criteria for LPN metastases and found that MRI had a sensitivity of 90%–100% irrespective of preoperative treatment.^13^ A number of other studies have also highlighted the sensitivity of diagnostic criteria based on the AUC in order to avoid missing LPN metastases.^30^, ^31^, ^32^, ^33^ On the other hand, we have developed diagnostic criteria that do not require use of an AUC‐based approach. Instead, we examined in detail the short diameter and reduction rate of LPNs to identify features of LPNs without residual metastases after the preoperative treatment. We consider the negative predictive value to be more important than sensitivity for accurately identifying patients in whom LPN dissection could be omitted. Although the choice of which indicators of diagnostic performance to use depends on the rationale used to develop the diagnostic criteria, the most common concept is keeping the false‐negative rate close to zero in order not to miss patients who need LPN dissection.

There are no reports that compared the effects of CRT and NAC on the short diameter of LPNs. In the present study, both CRT and NAC reduced the short diameter of LPNs and CRT tended to reduce it more than NAC. One possible reason for this is that CRT may have a greater antitumor effect than NAC. This higher antitumor effect may have resulted in a greater proportion of tumor cells disappearing or shrinking, leading to the diameter of the LPNs being smaller in the CRT group than in the NAC group. Furthermore, it is possible that irradiation may have caused shrinkage of normal lymph nodes as well as tumor cells. Although an influence of either of these two factors cannot be ruled out, they do not affect the diagnostic criteria for LPNs without residual metastases developed in this study.

The strength of this study lies in the fact that we were able to collect data from representative institutions that provide treatment for rectal cancer in Japan. Furthermore, MRI scans were acquired before and after preoperative treatment, and researchers at each institution measured the short and long diameters of LPNs on both MRI scans and on pathological examination. The LPNs were mechanically matched one‐to‐one based on size on pre‐ and posttreatment MRI scans and on pathological examination without knowledge of the pathological diagnosis. However, this study also has some limitations. LPN metastases were diagnosed based on pathological examination of specimens from patients in whom LPN dissection was performed after CRT or NAC. Therefore, it was not possible to determine whether CRT or NAC eliminated LPN metastases or whether LPNs without metastases after preoperative CRT or NAC were free of metastases before treatment. The degree of lymph node shrinkage may vary, and there may be a limit to the reliability as to whether or not they are actually the same lymph node because we only performed a mechanical match based on size. Because of the small number of LPNs with metastases, our criteria did not find a significant difference in the CRT group, although there were no metastases in LPNs that met our criteria. More patients in the CRT group underwent unilateral LPN dissection, whereas more patients in the NAC group underwent bilateral LPN dissection. This difference may have some influence on the diagnostic accuracy of this study. Only LPNs depicted by MRI were included. Therefore, the results of this study may not be applicable to cases in which LPN metastasis is suspected based on clinicopathologic factors other than MRI findings. Finally, we were unable to ascertain the prognosis of patients who did not actually undergo LPN dissection despite meeting our study's inclusion criteria.

## 5 CONCLUSION

Both CRT and NAC significantly reduced the short diameter of LPNs, but even LPNs with a short diameter of <5 mm could contain residual metastases in patients with rectal cancer. LPNs with a short diameter of <5 mm both before and after CRT or NAC that did not shrink after treatment showed no evidence of metastasis, suggesting that LPN dissection might be omitted in these cases.

## FUNDING INFORMATION

This study was funded by the Health Science Center.

## CONFLICT OF INTEREST STATEMENT

Authors declare no conflict of interests for this article.

## ETHICS STATEMENTS

Approval of the research protocol: The protocol for this research project was approved by the Ethics Committee of Kyoto University (Approval No. R1246).

Informed Consent: N/A.

Registry and the Registration No. of the study/trial: N/A.

Animal Studies: N/A.

## Supporting information


Table S1.

## DISCUSSANT

### Professor Yojiro Hashiguchi

Congratulations on your very fantastic and interesting presentation. It is a very unique investigation because the authors identified the lateral lymph nodes before and after CRT or NAC on a one‐to‐one correspondence on MRI, and clarified the relationship among the size, shrinkage of the lymph nodes, and the presence or absence of pathological lymph node metastasis. The presenter concluded that the MRI before and after treatment can identify the lateral lymph node without lymph node metastasis. To begin with, I would like to ask about some methodological aspects. On average, how many lateral lymph nodes did you identify per patient on a one‐to‐one basis?

How did you ensure that the lymph node identification was correct? Did you match the lymph nodes on MRI before and after treatment without knowing the pathology results?

How did you treat the lymph nodes that were visible on MRI before preoperative treatment and no longer visible after the treatment? In that case, would you consider that the invisible lymph node has shrunk due to the treatment?

### Dr. Nobuaki Hoshino Response

Thank you for your questions and comments. On average, 15 lateral lymph nodes were identified by pathological examination and four by MRI per patient. Therefore, an average of four lateral lymph nodes per patient were matched one‐to‐one to pre‐treatment MRI and post‐treatment MRI scans and pathological examination.

Researchers at each institution measured their own data for short and long diameters of lateral lymph nodes both on MRI and pathological examination. Without knowing the pathological diagnosis, the principal investigator at Tokyo University mechanically matched lymph nodes based on size so that they would have a one‐to‐one correspondence. Furthermore, we confirmed that matching was performed correctly based on size.

Lymph nodes that were no longer visible on MRI after preoperative treatment were considered to have decreased in size because of treatment and their short diameter was treated as 0 mm.

### Professor Yukihide Kanemitsu

Congratulations for your excellent work. You showed a figure of pre‐ and post‐treatment short diameter. How did you match between MRI and pathological specimen? That means you weren't looking to see if there were lymph nodes that had shrunk among the ones you had collected, checking if each lymph node had undergone change by comparing it before and after treatment? This means that you assessed shrinkage by using average values or something along those lines?

You mean the pre and post criteria of short diameter of lymph node must both be important in the context of omitting lateral lymph node dissection?

In such a case, I think sensitivity and negative predictive value must both be important to omit some procedures which cannot be determined by the AUC. In your study, what are the numbers of sensitivity and NPV?

### Dr. Nobuaki Hoshino Response

We appreciate your questions and observations. Pre‐treatment lateral lymph nodes on MRI, post‐treatment lateral lymph nodes on MRI, and lateral lymph nodes on pathological examination were mechanically matched based on size. Lateral lymph nodes were sorted by region and matched to provide a one‐to‐one correspondence. The degree of reduction in lymph node size may vary, and there may be a limit to reliability in terms of whether or not they are actually the same lymph node because we only performed a mechanical match based on size.

Some lymph nodes with a short diameter of 5 mm or less showed metastasis. We do not believe that metastasis necessarily disappears when lateral lymph nodes are reduced in size by preoperative treatment. Instead, we think that if there is no metastasis, there would be no reduction in size. This is why we came up with the idea of creating this diagnostic criterion.

The diagnostic criterion for lymph nodes without metastasis developed in this study did not use an AUC‐based method. Rather, we identified the characteristics of lymph nodes without metastasis by examining their short diameter and rate of reduction in detail.
