# Supplementary material for: Identification of lateral pelvic nodes without metastasis in patients with rectal cancer treated with preoperative chemoradiotherapy or chemotherapy based on magnetic resonance imaging
Source: Ann Gastroenterol Surg. 2024 Jun 1;8(5):732–9. doi: 10.1002/ags3.12832 (PMC11368503; doi:10.1002/ags3.12832)
Supplement: Supplementary file 1 — Table S1. [file AGS3-8-732-s001.docx]

| Table S1 Preoperative treatment regimens | | | | |
| --- | --- | --- | --- | --- |
| Chemoradiotherapy (n =28) | |  | Neoadjuvant chemotherapy (n = 47) | |
| Regimen* | n |  | Regimen | n |
| UFT/LV | 20 |  | SOX | 18 |
| TEGAFIRI | 3 |  | XELOX | 11 |
| Capecitabine | 2 |  | FOLFOX | 6 |
| IRIS | 2 |  | SOX-bevacizumab | 5 |
| S-1 | 1 |  | FOLFOX-panitumumab | 4 |
|  |  |  | XELOX-bevacizumab | 2 |
|  |  |  | FOLFOX-bevacizumab | 1 |
| FOLFOX : fluorouracil, leucovorin, and oxaliplatin | | | | |
| IRIS : S-1 and irinotecan | | | | |
| SOX : S-1 and oxaliplatin | | | | |
| TEGAFIRI : tegafur-uracil, leucovorin, and irinotecan | | | | |
| UFT/LV : tegafur-uracil and leucovorin | | | | |
| XELOX : oxaliplatin and capecitabine | | | | |
| *with 40-50.4Gy irradiation | | | | |
